# Long non-coding RNAs may serve as biomarkers in breast cancer combined with primary lung cancer

**DOI:** 10.18632/oncotarget.17356

**Published:** 2017-04-21

**Authors:** Xianfeng Ding, Yuhan Zhang, Hongjian Yang, Weimin Mao, Bo Chen, Shifeng Yang, Xiaowen Ding, Dehong Zou, Wenju Mo, Xiangming He, Xiping Zhang

**Affiliations:** ^1^ College of Life Science, Zhejiang Sci-Tech University, Hangzhou 310008, Zhejiang Province, China; ^2^ Department of Breast Surgery, Zhejiang Cancer Hospital, Hangzhou 310022, Zhejiang Province, China; ^3^ Department of Thoracic Surgery, Zhejiang Cancer Hospital, Hangzhou 310022, Zhejiang Province, China; ^4^ Department of Pathology, Zhejiang Cancer Hospital, Hangzhou 310022, Zhejiang Province, China

**Keywords:** lncRNA, breast cancer combined with primary lung cancer, biomarkers

## Abstract

Long non-coding RNAs (lncRNAs) have been shown to play important regulatory role in certain type of cancers biology, including breast and lung cancers. However, the lncRNA expression in breast cancer combined with primary lung cancer remains unknown. In this study, databases of the Cancer Genome Atlas (TCGA) and the lncRNA profiler of contained candidate 192 lncRNAs were utilized. 11 lncRNAs were differentially expressed in breast cancer, 9 candidate lncRNAs were differentially expressed in lung cancer. In order to find the aberrant expression of lncRNAs in breast cancer combined with primary lung cancer, seven samples of primary breast cancer and lung cancer were studied for the expression of selected lncRNAs. The results showed that SNHG6 and NEAT1 were reversely expressed in breast cancer combined with primary lung cancer compared with primary breast or lung cancer. In addition, a significant correlation of lncRNAs was found in the patients whose age was above 56 in breast cancer. What's more, PVT1 expression was negatively correlated with the pathological stage, and the level of ER, PR, HER2, p53 in breast cancer. Furthermore, lncRNA expression did not have significant relationship with the 5-year survival of patients with breast cancer combined with primary lung cancer. The findings revealed that PVT1, SNHG6, NEAT1 may serve as a prognostic marker for breast cancer combined with primary lung cancer. Therefore, these lncRNAs are potential molecular indicators in the diagnosis and prognosis of cancer in the future.

## INTRODUCTION

The human genome is the blueprint of the encrypted codes for protein synthesis. However, transcripts from the whole genome are scarcely (only about 2%) used for actual protein synthesis, most of transcripts are non-coding RNAs including microRNAs and long non-coding RNAs (lncRNAs) [1]. LncRNAs are more than 200 nucleotides in length, longer than microRNAs. According to genomic organization, lncRNAs can be classified into five broad categories: sense, antisense, bidirectional, intronic and intergenic [2]. Originally considered as “transcription noise”, lncRNAs have been reported to play important roles in cellular functioning and tumorigenesis through various mechanisms, including post-translational modification, post-translational inhibition and chromatin remodeling, etc [3]. In recent years, a large amount of studies proved that abnormal expression of lncRNAs such as H19 [4], MALAT1 [5], HOTAIR [6], PVT1 [7], may contribute to cancer development. However, the functions and molecular mechanism of lncRNAs is not clear. Neither is it known whether lncRNAs can serve as biomarkers for cancer diagnosis and prognosis.

Breast cancer is the most frequent malignancies in women worldwide in 2017 based on the latest report on American Cancer Society [8]. The statistics pointed out that breast cancer cases account for 30% of all new cancer cases and rank the second in mortality. Breast cancer is a heterogeneous disease with poor prognosis due to multiple genetic alterations [9]. Recently, several lncRNAs have been shown to serve as breast cancer biomarkers. For example, Roxana team evaluated the expression of lncRNA CCAT2 by reverse transcription-qPCR and *in situ* hybridization (ISH) in breast carcinoma tissue and adjacent tissues [10], which indicated that CCAT2 could be act as a novel biomarker to predict the clinical outcome. Similar pattern of statistics showed that more than 105510 new lung cases were added in 2017 [8], which is a leading cause of cancer death. Therefore, it is urgent to find new prognostic marker and therapeutic strategies to improve the treatment of lung cancer. PVT1 is known as an oncogene, some evidence has indicated that PVT1 is upregulated in non-small cell lung cancer tissue, and its upregulation is associated with lymph node metastasis [11]. Unlike microRNAs which have been vigorously studied, lncRNAs remains largely unknown.

In recent decades, along with the progress in early diagnosis and improvement of cancer treatment, overall survival time of cancer patients is prolonged significantly. However, longer survival time is accompanied with increased risk of multiple malignant primary tumors [12]. Breast cancer combined with primary lung cancer is a kind of multiple primary malignancies, and lung cancer is a common second primary malignancy developing in the patients who has a history of previous malignancy. Perng [13] analyzed 193 patients with multiple primary cancers, which showed that about 40% of patients were diagnosed with lung cancer before the occurrence of other primary cancers, and patients with lung cancer were at a higher risk of developing multiple primary neoplasms. Although lncRNAs may play an important role in breast cancer and lung cancer, little is known whether lncRNAs can serve as biomarkers in multiple primary neoplasms.

To better understand the roles of lncRNAs in breast cancer combined with primary lung cancer, comprehensive analysis of expression abundance of lncRNAs is imperative. In this study, some lncRNAs associated with breast cancer and lung cancers were chosen by using TCGA at cBioportal and lncRNA profiles. The results revealed that a few lncRNAs were aberrantly expressed in eleven breast cancer combined with primary lung cancer tissues compared with seven primary breast cancer or lung cancer tissues. Furthermore, the correlation between pathological parameters and lncRNA aberrant expression were analyzed. This study provided the framework for future research on lncRNAs in breast cancer combined with primary lung cancer.

## RESULTS

### Initial screening results of lncRNAs

**Figure 1:**
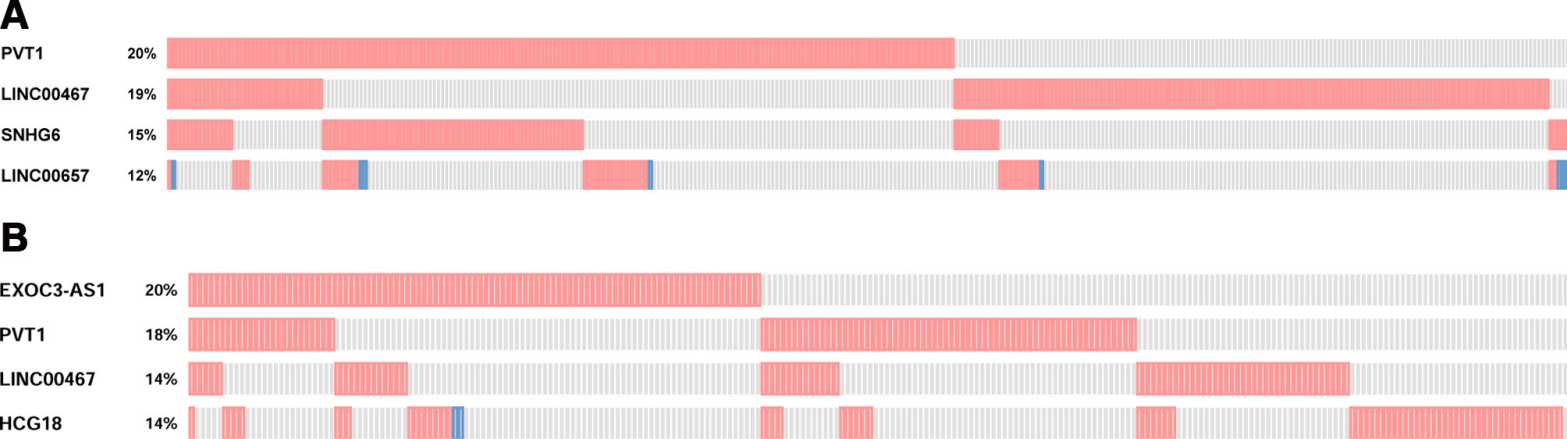
(**A**) Top 4 lncRNAs differentially expressed in breast cancer based on TCGA. (**B**) Top 4 lncRNAs differentially expressed in lung cancer based on TCGA.

To select lncRNAs with differential expression, we screened the dataset of the Cancer Genome Atlas (TCGA). In this study, we selected four lncRNAs which had expression alterations ranging from 12% to 20% in breast carcinoma, including PVT1, linc00467, SNHG6, linc00657 (Figure 1A). As shown in Figure 1B, we also selected four lncRNAs associated with lung cancer. Among them, EXOC3-AS1 had the highest frequency (21%), the others were PVT1, linc00467, HCG18. In addition, we set parameter RNA Seq V2 RSEM when selected genomic profiles before submitted. In summary, a total of 192 lncRNAs from lncRNAs profilers were tested in breast cancer and lung cancer tissues. The results showed that lncRNAs AFAPA-AS1, aHIF, BDNF-AS, HYMAI, UCA1, kucg 1, MALAT1 were differentially expressed in breast cancer tissues (fold change > 2). In the differentially expressed lncRNAs, BANCR, kucg 1, MALAT1, NEAT1 were chosen for further study in lung cancer. All of them were performed to verify the eleven breast cancer combined with primary lung cancer RNA samples. The results of lncRNA microarray were showed in Supplementary Tables 1, 2, 3, 4.

**Table 1:** Clinic pathologic characteristics of patients with experiment samples

Variable	Clinic pathologic parameter	Number of cases
Case		25
	Multiple primary neoplasms	11
	Primary breast cancer	7
	Lung cancer	7
Age	Range	38–69
Therapy	Chemotherapy	0
	Radiotherapy	0
	no	25
Histological grade (breast cancer combined with primary lung cancer)	I	2
	II	7
	III	1
	IV	1
ER	negative	7
	positive	11
PR	negative	8
	positive	10
HER2	negative	13
	positive	5
P53	negative	9
	positive	9
TTF1	negative	0
	positive	11
	unknown	7
Napsin A	negative	0
	positive	11
	unknown	7
P63	negative	4
	positive	7
	unknown	7
Ck7	negative	0
	positive	11
	unknown	7

### Measurement of lncRNAs expression in breast and lung cancers tissues by qRT-PCR

**Figure 2:**
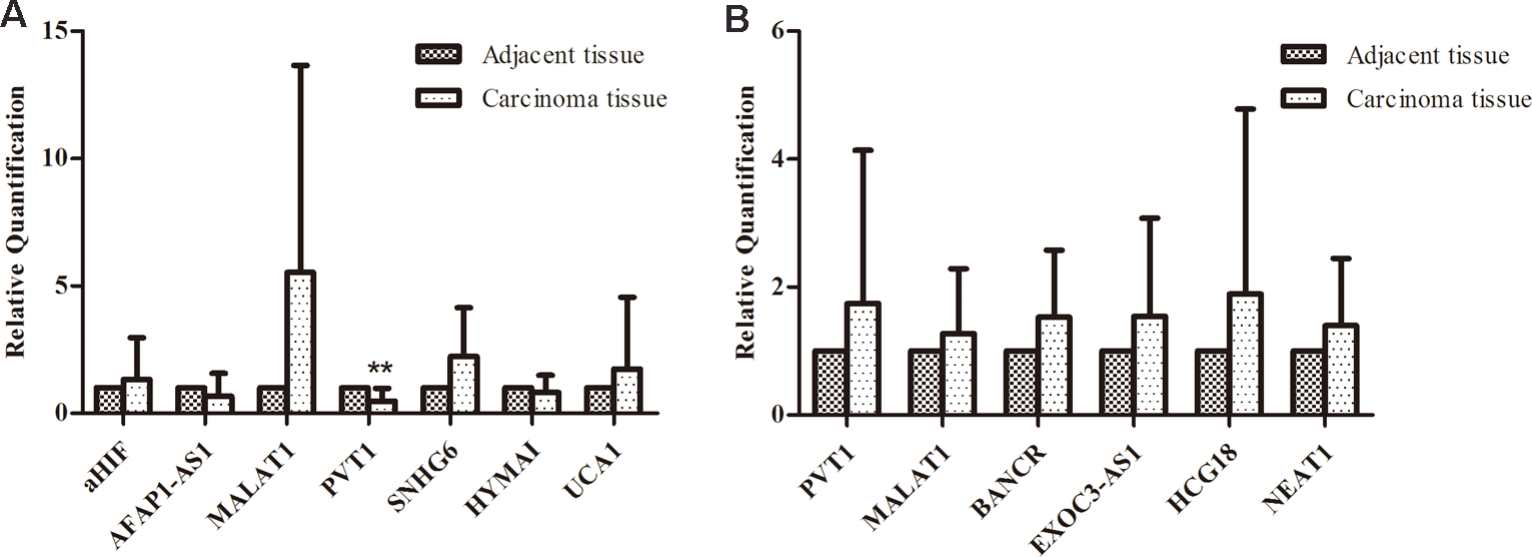
Comparison of lncRNAs aberrant expression in multiple primary neoplasms and their counterpart adjacent tissue (**A**) The expression of selected lncRNAs in breast cancer tissues using qRT-PCR; (**B**) Comparison of selected lncRNAs aberrant expression in lung cancer tissues with matched adjacent tissues to cancer.)

The expression of selected lncRNAs was analyzed in 11 breast cancer tissues and their counterpart adjacent tissues. In this study, lncRNAs were aberrantly expressed in breast cancer tissues either by amplification or by upregulation. As shown in Figure 2A, aHIF, MALAT1, SNHG6, UCA1 expression levels increased in breast cancer tissues compared with their counterpart adjacent tissues, while AFAP1-AS1, PVT1, HYMAI had lower expression in tumors. Another lncRNAs associated with breast cancer had no significant difference from the control. We then analyzed lncRNAs expression in lung cancer samples by qRT-PCR. Compared with adjacent tissues, PVT1, HCG18, EXOC3-AS1 were upregulated in most lung cancer tissues (Figure 2B). Due to individual variation in different samples and the clinical specimens, we calculated the sample average as mean ± SD and examined the trend of these genes expression.

**Figure 3:**
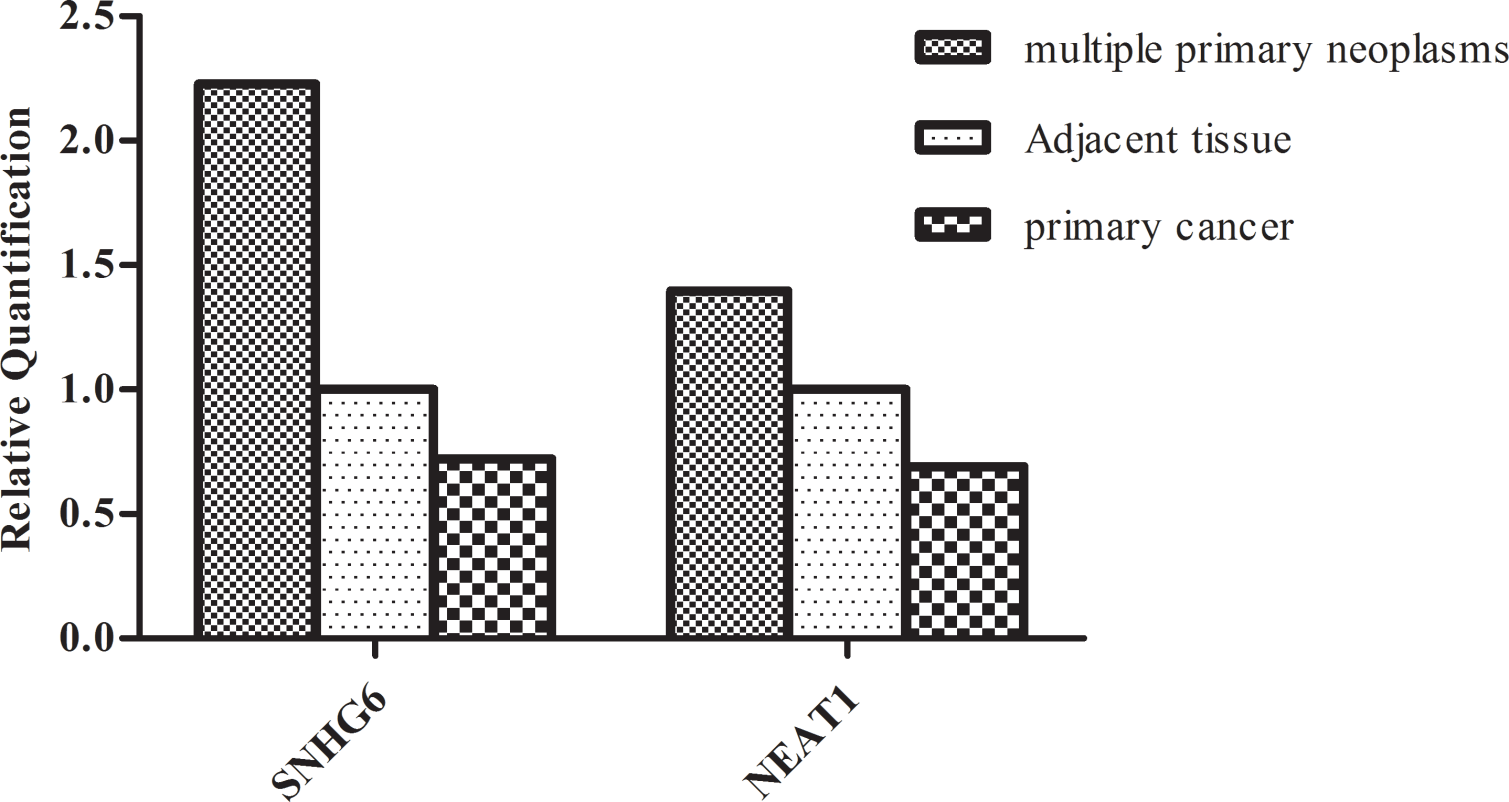
Comparison of lncRNAs (SNHG6,NEAT1) aberrant expression in multiple primary neoplasms with primary cancer Compared with adjacent tissues, SNHG6 was up-regulated in most breast cancer tissues while down-regulated in primary breast cancer tissues. NEAT1 was down-regulated in primary lung cancer tissues but had higher levels of expression in lung cancer. The data indicated that SNHG6 and NEAT1 could be potential molecular indicators in breast cancer combined with primary lung cancer.)

To further determine the biomarkers in breast cancer combined with primary lung cancer, we validated some lncRNAs in primary breast cancer and lung cancer tissues. Some lncRNAs were studied in primary breast cancer or lung cancer and their expression levels were consistent with our results. No further tests were done to these lncRNAs. For example, previous studies demonstrated that MALAT1 was upregulated in lung cancer [14] and breast cancer [15]. As shown in Figure 2A, the consistent differential expression suggested that MALAT1 could potentially act as an oncogene in our breast cancer tissues. We evaluated the expression of lncRNA BNNF-AS, linc00467, SNHG6 in seven primary breast cancer tissues. The statistical significance data suggested that lncRNA BDNF-AS linc00467 were downregulated in primary breast cancer, whereas, they showed no significant change in breast cancer tissues compared with adjacent tissues in multiple primary neoplasms. While BDNF-AS (RQ = 0.5950, *P* = 0.07) played the role of primary breast cancer inhibitor. As shown in Figure 3, lncRNA SNHG6 was downregulated in primary breast cancer while upregulated in breast cancer combined with primary lung cancer. A previous study confirmed that SNHG6 aberrantly expressed in human would result in cancer [16]. The results in this study suggested that SNHG6 upregulation could potentially cause human breast carcinogenesis with multiple primary neoplasms. Using the same method, the expression of lncRNAs MALAT1, BANCR, NEAT1 in primary lung cancer were also analyzed. The results showed that MAlAT1 and BANCR had similar trend of expression between the two kinds of cancers. However, lung cancer had higher NEAT1 expression, when compared with primary lung cancer. The data indicated that NEAT1 overexpression might contribute to the incidence of breast cancer combined with primary lung cancer incidence. In fact, BANCR were shown to play a critical role in the proliferation and metastasis of malignant melanoma and lung cancer. lncRNA BANCR also played an important role in retinoblastoma aggressiveness. It is useful for therapeutic strategy and prognostic prediction [17]. MALAT1 also served as a marker to predict cancer progression, including multiple myeloma, lung cancer, and cervical cancer [14]. NEAT1, a novel lncRNA, is potentially an important regulator in several kinds of cancers. For example, NEAT1 might be associated with tumorigenesis and progression in non-small cell lung cancers [18]. Convincing evidence also showed that NEAT1 drove oncogenic growth in prostate cancer [19]. Our study suggested that, NEAT1 may be used as a prognostic parameter for breast cancer combined with primary lung cancer.

### LncRNAs expression in breast cancer associated with clinical pathologic feature

The overexpression of ER and PR was proved to be important in breast cancer oncogenesis. A vast variety of researches showed that estrogen receptor (ER), progesterone receptor (PR), human epidermal growth factor receptor 2 (HER2), and EGFR presence uncovered by immunohistochemistry (IHC) is associated with cancer cell differentiation or development. They are useful indicators in endocrine therapy in breast cancer [20]. In this study, the correlation between lncRNA and clinical pathologic features, including ER/PR, HER2, and p53 protein expression were analyzed. The expressions of the lncRNAs aHIF, BDNF-AS, linc00467, SNHG6, HYMAI, UCA1 were unrelated to the expression of ER or PR. Only AFAP1-AS was significantly downregulated in ER-, PR- patients (*P* = 0). PVT1 was significantly downregulated in ER+ and PR- patients (*P* = 0.011, *P* = 0).

**Figure 4:**
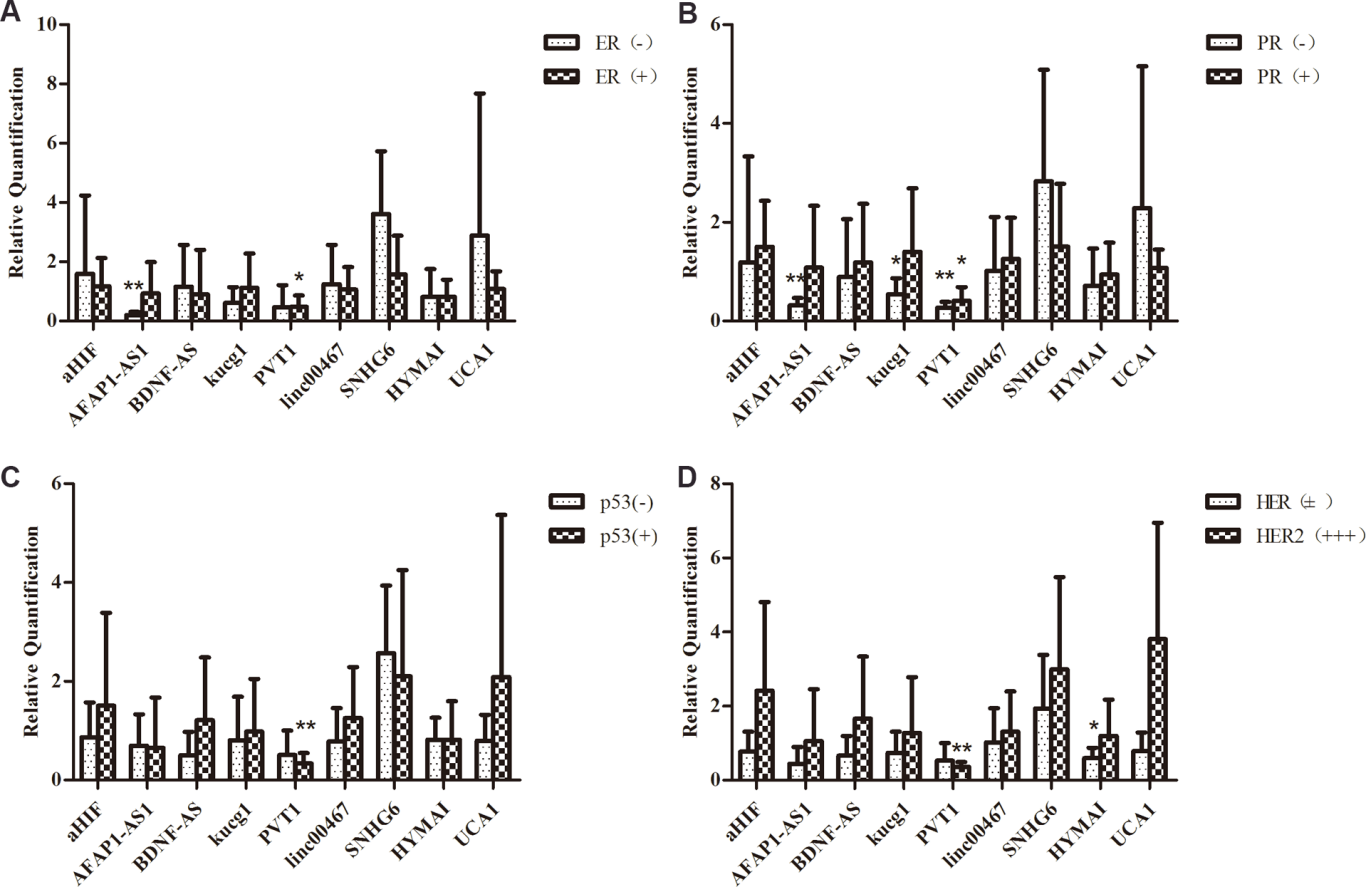
Comparison of lncRNAs aberrant expression in breast cancer with multiple primary neoplasms tissues with matched adjacent tissues in the level of clinic pathological (**A**)The expression levels of ER and PR (**B**), p53 (**C**) and HER2 (**D**).

**Figure 5:**
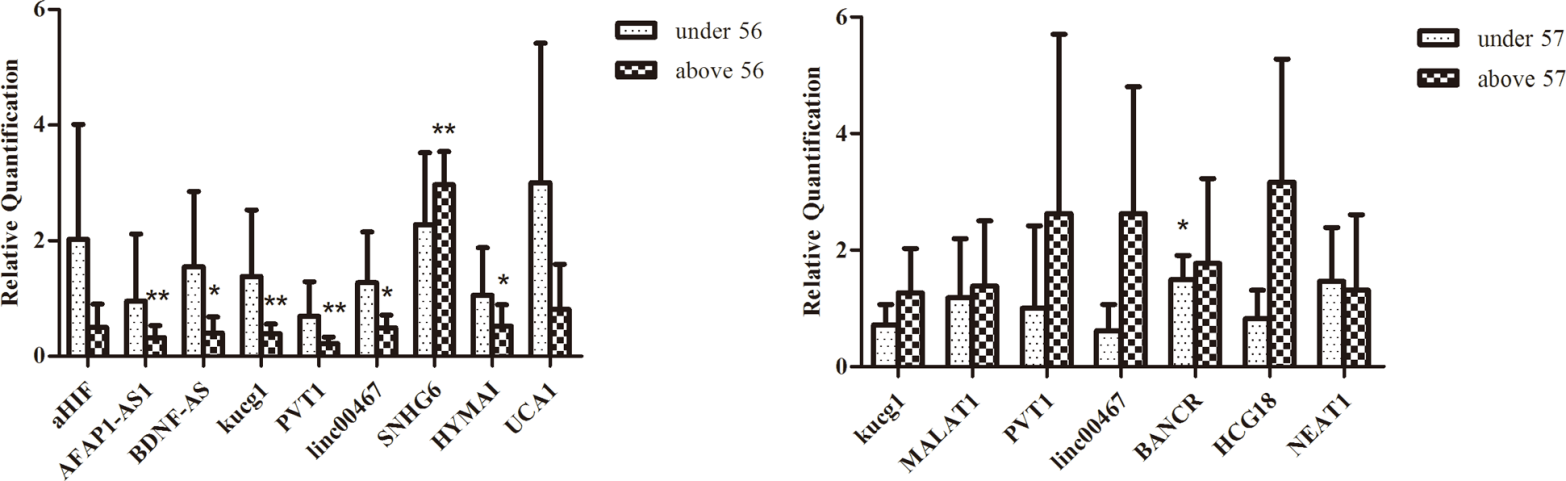
Comparison of lncRNAs aberrant expression in breast cancer combined with primary lung cancer in patients’ age (**A**) Comparison of lncRNAs expression in breast cancers with matched adjacent tissues on the level of age (> 56 and ≤ 56). (**B**) Comparison of lncRNAs expression in lung cancers with matched adjacent tissues on the level of age (> 57 and ≤ 57).

HER2 is a major target gene in breast cancer therapy [21], and p53 is an active tumor suppressor [22]. While most of the lncRNAs expression were not significantly altered among HER2 negative and HER2 positive breast cancer tissues in our study, PVT1 showed significantly lower expression in HER2 positive and p53 positive patients (*P* = 0.001, *P* = 0.006)(Figure 4). In most cases, HER2 positive (HER2+++) and P53 positive (p53+) breast cancers were accompanied by a poor clinical prognosis. Studies have suggested that overexpression of PVT1 induced the breast cancer invasiveness, lymph node metastasis [7]. It is speculated that PVT1 downregulation could result in decreased breast cancer cells migration, metastasis and proliferation in breast cancer combined with primary lung cancer. Breast cancer patients with longer survival time may have time to develop another primary cancer. The patients in this study were divided into two groups above and under their median age [23]. LncRNAs BDNF-AS, AFAP1-AS1, kucg1, PVT1, HYMAI downregulated in above 56 years, and SNHG6 was upregulated in breast cancer, while they were not significantly different in younger group (≤ 56). Meanwhile, the expression of lncRNAs kucg1, MALAT1, PVT1, linc00467, HCG18, NEAT1 did not show statistically significant difference in lung cancer tissues, except for BANCR (*P* = 0.033) which was upregulated in patients under 57 (Figure 5).

The qPCR data results indicated that lncRNAs expression had a positive correlation with carcinogenesis. Breast cancer combined with primary lung cancer may be more likely to happen in the patients older than 56 years.

### Prediction of interaction between lncRNAs and cancer associated protein

**Figure 6:**
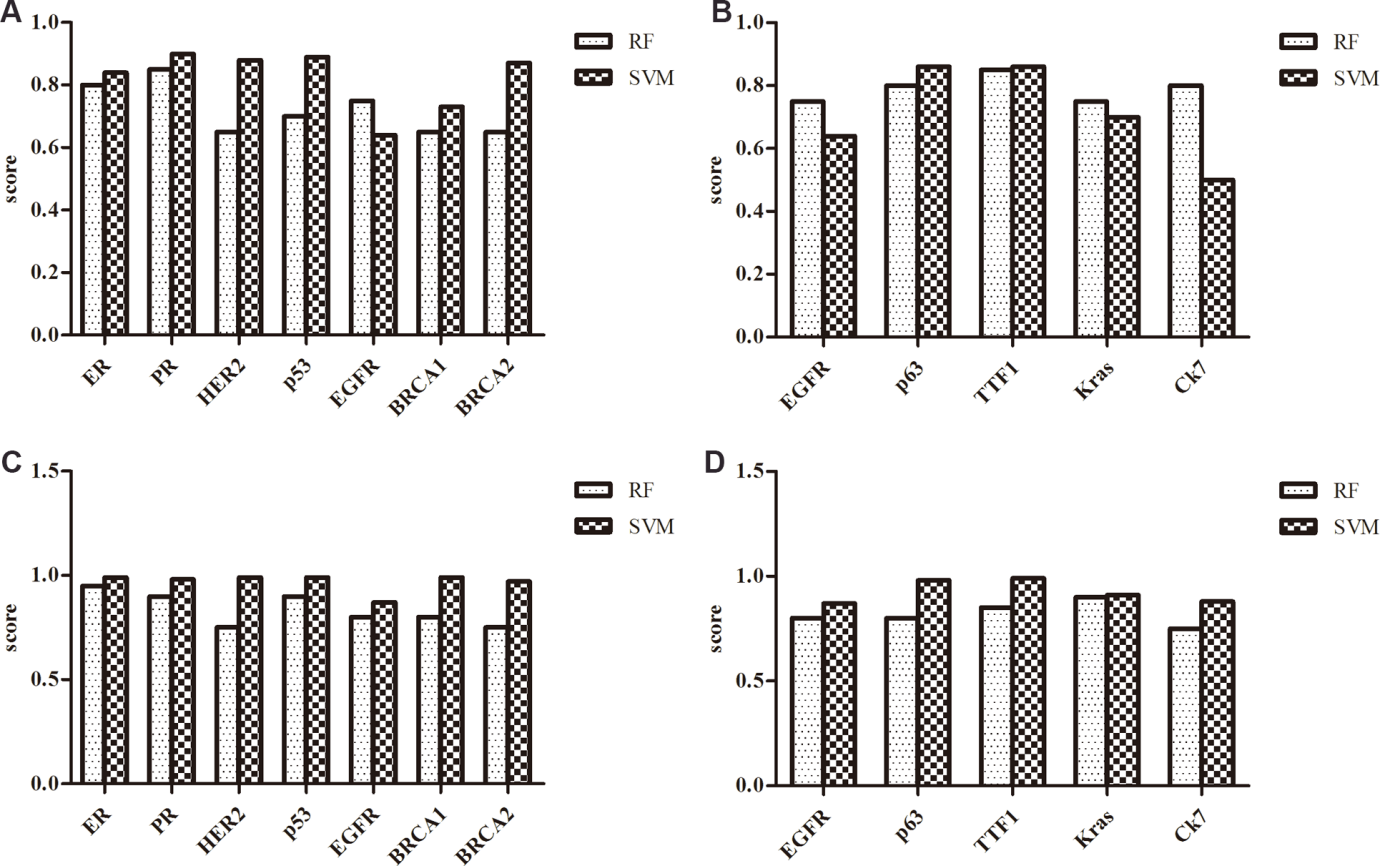
The scores of the interaction probability between lncRNAs and breast cancer or lung cancer associated protein predicted by RPISeq (**A**) The scores of the interaction probability between PVT1 and breast cancer associated proteins. (**B**) The scores of the interaction probability between PVT1 and lung cancer associated proteins. (**C**) The scores of the interaction probability between MALAT1 and breast cancer associated proteins. (**D**) The scores of the interaction probability between MALAT1 and lung cancer associated proteins.

RNA-protein interactions were proven to be very important in transcriptional and post-transcriptional regulation of gene expression. Identifying novel RNA-protein interactions will add valuable information about the RNA-protein interaction networks. RPISeq, a bioinformatics method, can be used to predict RNA-protein interactions by using sequence analysis [24]. (http://pridb.gdcb.iastate.edu/RPISeq/). RPISeq contained Random Forest (RF) and Support Vector Machine (SVM). In addition to clinical pathologic features, BRCA1 and BRCA2 are functional proteins associated with breast cancer. Likewise, TTF1, p63, NapsinA, K-ras, EGFR are associated with the oncogenesis of lung cancer. The results in this study showed that all lncRNAs selected had great possibility of interaction with breast cancer associated proteins. For example, PVT1 was predicted to interact with ER by RF (score = 0.8) and SVM (score = 0.84). Detail results were shown in Figure 6. In lung cancer, all selected lncRNAs were predicted to interact with functional protein having scores of RF or SVM bigger than 0.5. It was suggested that interaction between lncRNAs and some functional proteins may contribute to the tumorigenesis.

### Alterations of lncRNAs expression and prognosis in breast cancer combined with primary lung cancer

**Figure 7:**
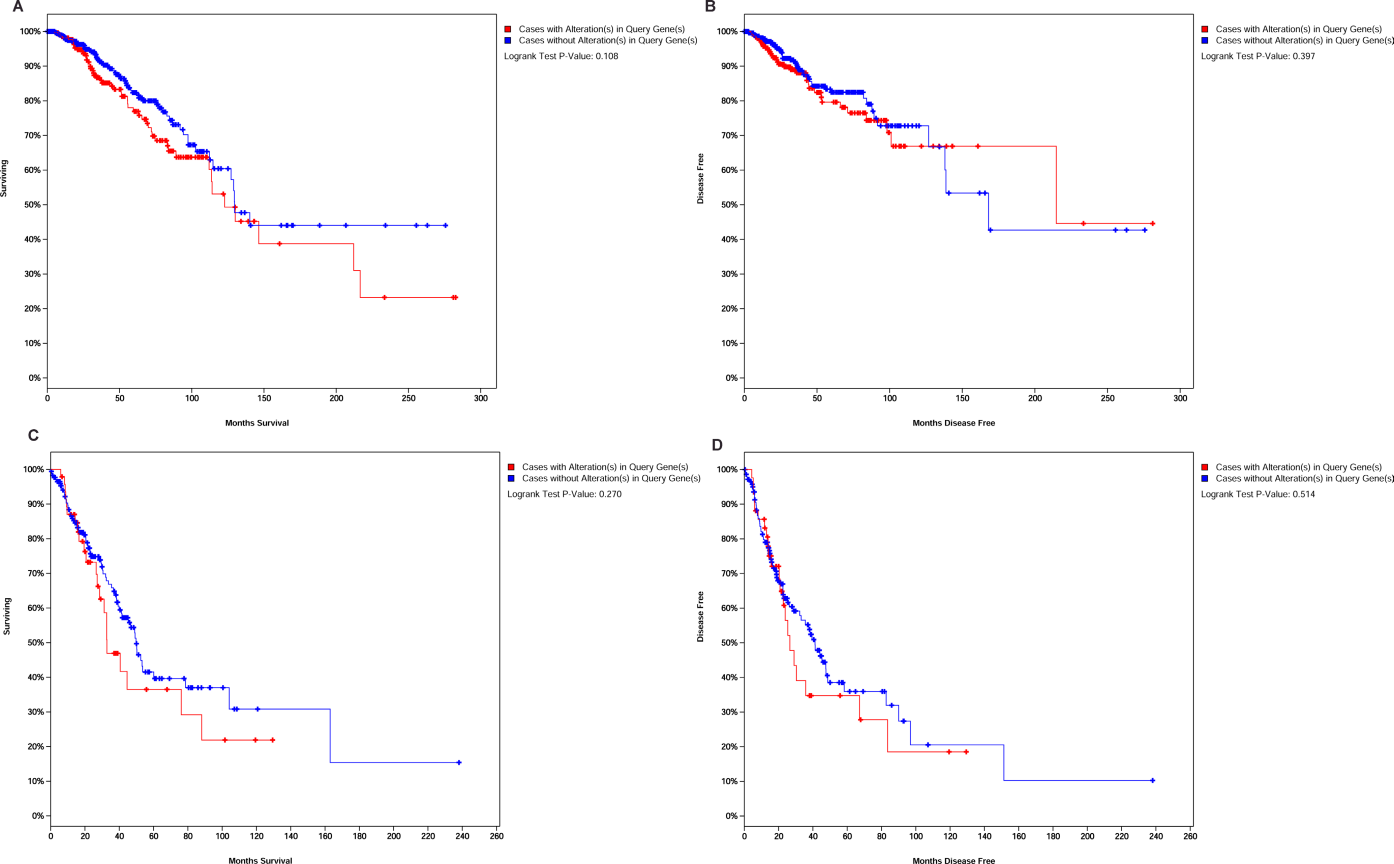
LncRNAs alteration associated with overall survival and disease free survival (**A**–**B**) Kaplan-Meier curve based on alteration of PVT1 and SNHG6 in breast cancer. (**C**–**D**) OS and DFS associated with alterations of PVT1 and NEAT1 in lung cancer.

To further study whether lncRNAs expression can be used to predict survival outcomes, PVT1 and SNHG6 were searched in TCGA at cBioportal. The *p* values for the two lncRNAs associated with OS and DFS were shown in Figure 7A–7B. It was shown that patients had lower five years survival rate in breast cancer with alteration in PVT1 and SNHG6. Meanwhile, alterations of PVT1 and NEAT1 will reduce the overall survival and disease free survival in lung cancer (Figure 7C–7D). This was consistent with our results and suggested that these lncRNAs might act as prognostic markers in breast cancer combined with primary lung cancer.

We also analyzed the correlation between lncRNAs expression and the cancer stage. LncRNA PVT1 was lower in grade I and III breast cancers, and SNHG6 was upregulated in grade I breast cancer. The data indicated that SNHG6 might be related to pathogenesis and early diagnosis of breast carcinoma, and PVT1 was likely to inhibit breast cancer aggressiveness. According to the information obtained from the case management system in the medical record room of the Zhejiang Cancer Hospital, it was found that the lung cancer was always in grade I in the cases of breast cancer combined with primary lung cancer. Lung cancer often happened as a second primary malignancy along with multiple primary neoplasms, which increase the prevalence and influence the survival time [25]. However, it is yet unclear whether lncRNAs expression affects the patients’ survival. The recalling survey from the 11 multiple primary neoplasms patients suggested that the majority of patients were still alive. Other studies found that having the history of a previous malignancy did not increase the risk of incomplete resection, and these multiple primary carcinomas could increase the 5-year survival rate compared with patients suffering from a first lung primary tumor [26]. This is consistent with our results. Moreover, a great proportion of patients with lung cancer as a second primary malignancy were in Stage I in our samples. Early diagnosis contributed to successful treatment. Meanwhile, the patients with multiple primary neoplasms might be closely followed up and given curative treatments in time.

## DISCUSSION

In recent years, lncRNA differential expression profiles in various cancers have been shown by microarray studies, RNA-Seq, and quantitative reverse transcription PCR (qRT-PCR). It is therefore suggested that lncRNAs might be used as potential biomarkers for cancer diagnosis and prognosis. Our previous study [23] made a comprehensive analysis of lincRNAs in breast cancer and matched adjacent tissue by RNA-Seq. The results suggested that aberrantly expressed lincRNAs might affect the development and progression of breast cancer. As a new method for gene microarray, Arraystar Human lncRNA Microarray version 2.0 was designed for the global profiling of human lncRNAs and protein-coding transcripts [27]. Some researchers performed chromogenic *in situ* hybridization (CISH) to identify lncRNAs expression [28]. In this study, lncRNAs were chosen based on lncRNAdb, lncRNAdisease and the Cancer Genome Atlas (TCGA). LncRNAdb contained a comprehensive list of lncRNAs and each entry included referenced information [29], such as sequences, structural information, expression, conservation, functional evidence and other relevant information. Similarly, LncRNADisease database developed a bioinformatic method to predict the lncRNAs relationship with diseases [30], especially cancer. We also used the database of TCGA at the cBioportal to preliminary screening of lncRNAs. The Cancer Genome Atlas (TCGA) at cBioportal is an open platform for exploring multidimensional cancer genomics data [31], providing access to molecular profiles and clinical attributes. Compared with RNA-Seq and gene microarray, lncRNA databases provided a more intuitive and convenient approach to explore the cancer genome data, and avoided the complicated experiment to directly min specific information from database.

A great amount of lncRNAs have already been revealed to correlate with various disease processes, including carcinogenesis. Breast cancer is a heterogeneous disease with a poor prognostic. Lung cancer, a leading cause of cancer-related mortality throughout the world, has a higher incidence. But the the survival and prognosis are unknown in breast cancer combined with primary lung cancer. Several studies showed that lncRNAs were potential prognostic markers in human breast cancer or lung cancer. For example, lncRNA PVT1 were reported to be dysregulated and likely played important roles in a variety of cancers. PVT1 could be a novel biomarker for diagnosis and prognosis of non-small cell lung cancer [32]. Moreover, lncRNA UCA1, an oncogene in breast cancer, affects breast cancer cell growth and apoptosis through downregulation of mir-143 [33]. MALAT1 is a type of long non-coding RNA associated with lung cancer and breast cancer. Previous studies prove that MALAT1 promotes migration, metastasis and proliferation of lung cancer. It also regulated migration and invasion of breast cancer cells through binding of miR-1 competitively [34]. In this study, lncRNA profilers and TCGA database were used to detect the lncRNAs expression in 11 paired breast cancer combined with primary lung cancer samples. Compared with breast adjacent tissues, AFAP1-AS1, PVT1, HYMAI were downregulated in most breast cancer tissues, and lncRNA PVT1, BANCR, HCG18 were upregulated in lung cancer. Zeng and his team members [35] found that AFAP1-AS1 was significantly upregulated in lung cancer and was associated with poor prognosis. Knockdown the AFAP1-AS1 inhibited cell invasion and migration capability in lung cancer cells. While the relationship between AFAP1-AS1 and breast cancer is not clear, this study provided some useful insight.

We further examined the expression of selected lncRNAs in primary breast cancer and lung cancer tissues. The results demonstrated that SNHG6 and NEAT1 had opposite change of expression levels in breast cancer combined with primary lung cancer patients. As an oncogenic lncRNA, PVT1 [36] induced proliferation and suppressed apoptosis in *in vitro* experiments. Moreover, PVT1 expression was significantly correlated with clinical features, such as risk, recurrence, and survival in various cancers [37]. However, PVT1 was significantly downregulated in breast cancer tissues when multiple primary neoplasms were present. It was speculated that downregulation of PVT1 decreased the breast cancer aggressiveness and induced the risk of second malignancies. In summary, these lncRNAs may serve as biomarkers for breast cancer combined with primary lung cancer. Our study provided a new insight into this novel type of multiple cancers.

The incidence of multiple primary malignancies has increased in recent decade, therefore it is particularly important to determine the clinical characteristics and prognosis. In the present study, we analyzed the clinical pathology features in breast cancer with multiple primary neoplasms. Owing to the facts that a great proportion of patients were in Stage I of lung cancer, it is not necessary to analyze lncRNA expression in lung cancer tissues with different tumor grade. We analyzed the lncRNAs expression in relationship with ER, PR, HER2, p53 and patients’ age. PVT1 was downregulated in ER+,PR+,HER2+,p53+. Thus the expression of these molecules could be predicted through the measurement of PVT1 expression in breast cancer. Wu [38] used an integrated systematic bioinformatics approach to explore the estrogen receptor (ER)-regulated transcriptome. The results suggested that the identification of lncRNAs expression in presence of ER may be useful in clinical treatment strategy and prognosis. Furthermore, lncRNA BDNF-AS, AFAP1-AS, kucg1, PVT1, linc00467, SNHG6, HYMAI were differently expressed in patients’ age above 56 years of age. This could be the reason of increased incidence of breast cancer in the elder patients. However, the lncRNAs expression was negatively correlated with patients’ age in lung cancer.

Indeed, It was suggested that the interactions existed between lncRNAs and cancer associated proteins including ER, PR, HER2, p53, BRCA1, BRCA2, p63, K-ras, EGFR, and TTF1. HER2 is a member of the epidermal growth factor receptor family and its amplification or overexpression play an important role in the development and progression of certain types of aggressive breast cancer. Mutations in the breast cancer genes BRCA1 and BRCA2 increase the risks to breast cancer, and BRCA1 and BRCA2 proteins act as tumor suppressor genes. Currently, ER, PR, HER2 and p53 were routinely used as indicators for breast cancer treatment and prognosis prediction [39]. In addition, the interaction between lncRNAs and lung cancer associated proteins were suggested. EGRF is a potent mitogenic factor in a variety of cells, promoting epithelial cell proliferation. EGFR mutations were found in lung cancer. Alexander reported that patients with malignant pleural effusion of lung adenocarcinoma had increased frequency of EGFR and KRAS mutations [40]. TTF1 is a transcription termination code. The overexpression of TTF1 was associated with poor prognosis in patients with colorectal cancer [41]. P63 induced expression of many p53-target genes that were involved in cell-cycle arrest and apoptosis, so p63 is useful in distinguishing non-small cell lung carcinoma patients with or without EGFR/KRAS mutation [42]. In this study, we found RPISeq offered a convenient and inexpensive method for computational construction of RNA-protein interaction networks, and provided useful insights into the function of long non-coding RNAs.

A group of lncRNAs were differently expressed in breast cancer combined with primary lung cancer, featured by either downregulation or upregulation. It was suggested that they might act as oncogenes or tumor suppressors in the development and progression of cancer. This study provided a new insight for the prediction of the prognosis of multiple primary neoplasms. However, there were still limitations in our study. For example, it was hard to collect enough tissues with multiple primary neoplasms. It was important to collect and study a larger amount of samples. In addition, it was not possible to obtain the cell line of breast cancer combined with primary lung cancer. Further studies need to be done to explore the lncRNA function and molecular mechanism. RNA Knock-Out and western blotting could be used in the future.

## MATERIALS AND METHODS

### Tissue samples and clinical information

FFPE tissues from twenty-five women were obtained from Zhejiang Cancer Hospital (during 2010 to 2015), 11cases of multiple primary neoplasms contained breast cancer tissues, lung cancer tissues and their counterpart adjacent tissue. 7 cases of primary breast cancer tissue and 7 cases of primary lung cancer tissue were used as control. All samples were collected with informed consent from the patients. The data analysis was kept anonymously. No patients received chemotherapy or radiotherapy before the samples were collected. FFPE tissues were stored in 4°C before processing. Clinical and pathological parameters of breast cancer included ER, PR, HER2, p53 and the age of patients. For patients with lung cancer information of CK5/6, ALK-NC, p63, Napsin A, TF1, CK7 and the age of patients were collected. Patient features were described in Table 1. Clinical stage of cancer were based on AJCC staging system [43].

### Selecting differentially expressed lncRNAs in breast cancer combined with primary lung cancer

### LncRNA profiler

In order to select the differently expressed genes in multiple primary neoplasm via bioninformatics approach, we inquired the breast invasive carcinoma and lung carcinoma dataset of the Cancer Genome Atlas TCGA) at the cBioportal (http://www.genenames.org/). Then, the cancer study and genomic profiles were submitted for further analysis. Finally, decoding data and sorting the lncRNAs expression were done.

In this study, we also selected differentially expressed lncRNAs by lncRNA profiler provided by University of Mississippi Medical Center. There were a total of 192 lncRNAs associated with cancer in plate 1 and plate 2. LncRNAs in plate 1 were from lncRNAdb (http://www.lncrnadb.org/). LncRNAs in plate 2 were from the lncRNAdisease (http://www.cuilab.cn/lncrnadisease).

Total RNA was isolated from samples tissues by using RecoverAll^TM^ Total Nucleic Acid isolation kit for FFPE according to factory protocol. RNA purity was measured by Nanodrop-2000. The A260/A280 in each RNA sample was above 1.8 and A260/A230 above 2.0.

Total RNAs isolated from four patients in breast cancer combined with primary lung cancer were mixed using the next qRT-PCR. The cDNA was synthesized using Reverse Transcriptase M-MLV form Vazyme Biotechnology. Each real-time PCR reaction (in 10 μl) contained 5 μl 2×ChamQ SYBR Green qPCR Master Mix (High Rox Premixed) (Vazyme), 1 μl primer (10 μM ) and 1 μl cDNA. The cycling conditions consisted of an initial denaturation, 95°C for 5 min, followed by 40 cycles at 95°C,15 sec and 60°C, 30 sec. The last stage is 95°C for 15 sec, 60°C for 1 min and 95°C for 15 sec. In addition, qPCR procedures were run in the StepOnePlus^TM^ and the relative quantification of gene expression was evaluated by ΔΔCT method [44]. We selected the threshold values of ≥ 2 or ≤ –2 fold change.

### Identifying significant differentially expressed lncRNAs in breast cancer combined with primary lung cancer

qRT-PCR was performed to determine gene expression in the samples. Based on standard protocols, the first strand cDNA was synthetized with M-MLV (Vazyme, Nanjing, China). The qPCR reaction conditions were same as previously described. PCR amplification was performed in triplicates for each sample, and glyceraldehyde-3-phosphate dehydrogenase (GAPDH) was used as an internal gene reference. The primer information of lncRNAs for qPCR was shown in Supplementary Table 5.

### Data processing and analyzing

In order to reduce errors, every independent RNA sample was performed in triplicate. Statistical calculations were performed with the SPSS 17.0, *P* values less than 0.05 were considered statistically significant when independent samples *t*-test was used.

### Bioinformatics analysis

In order to more deeply explore the relationship between clinical pathologic feature and lncRNAs in breast and lung cancers, computer analysis based on RNA-Protein interaction prediction (RPISeq) program, which used both random forest (RF) and support vector machine (SVM) classifiers were utilized to predict the interaction between lncRNAs and breast cancer and lung cancer associated proteins. The interaction probabilities generated by RPISeq range from 0 to 1. If the predictions with probabilities were > 0.5, we considered them positive, indicating that the corresponding RNA and protein were likely to interact.

## SUPPLEMENTARY MATERIALS TABLES











## References

[1] Kung JT, Colognori D, Lee JT (2013). Long noncoding RNAs: past, present, and future. Genetics.

[2] Ponting CP, Oliver PL, Reik W (2009). Evolution and functions of long noncoding RNAs. Cell.

[3] Johnsson P, Lipovich L, Grander D, Morris KV (2014). Evolutionary conservation of long non-coding RNAs; sequence, structure, function. Biochimica et biophysica acta.

[4] Li H, Yu B, Li J, Su L, Yan M, Zhu Z, Liu B (2014). Overexpression of lncRNA H19 enhances carcinogenesis and metastasis of gastric cancer. Oncotarget.

[5] Yoshimoto R, Mayeda A, Yoshida M, Nakagawa S (2016). MALAT1 long non-coding RNA in cancer. Biochimica et biophysica acta.

[6] Gökmen-Polar Y, Vladislav IT, Neelamraju Y, Janga SC, Badve S (2015). Prognostic impact of HOTAIR expression is restricted to ER-negative breast cancers. Sci Rep.

[7] Cui M, You L, Ren X, Zhao W, Liao Q, Zhao Y (2016). Long non-coding RNA PVT1 and cancer. Biochem Biophys Res Commun.

[8] Siegel RL, Miller KD, Jemal A (2017). Cancer Statistics, 2017. CA Cancer J Clin.

[9] Meng J, Li P, Zhang Q, Yang Z, Fu S (2014). A four-long non-coding RNA signature in predicting breast cancer survival. J Exp Clin Cancer Res.

[10] Redis RS, Sieuwerts AM, Look MP, Tudoran O, Ivan C, Spizzo R, Zhang X, de Weerd V, Shimizu M, Ling H, Buiga R, Pop V, Irimie A (2013). CCAT2, a novel long non-coding RNA in breast cancer: expression study and clinical correlations. Oncotarget.

[11] Liu FT, Xue QZ, Zhu ZM, Qiu C, Hao TF, Zhu PQ, Luo HL (2016). Long noncoding RNA PVT1, a novel promising biomarker to predict lymph node metastasis and prognosis: a meta-analysis. Panminerva Med.

[12] Mariotto AB, Rowland JH, Ries LA, Scoppa S, Feuer EJ (2007). Multiple cancer prevalence: a growing challenge in long-term survivorship. Cancer Epidemiol Biomarkers Prev.

[13] Liu YY, Chen YM, Yen SH, Tsai CM, Perng RP (2002). Multiple primary malignancies involving lung cancer-clinical characteristics and prognosis. Lung Cancer.

[14] Cho SF, Chang YC, Chang CS, Lin SF, Liu YC, Hsiao HH, Chang JG, Liu TC (2014). MALAT1 long non-coding RNA is overexpressed in multiple myeloma and may serve as a marker to predict disease progression. Bmc Cancer.

[15] Huang NS, Chi YY, Xue JY, Liu MY, Huang S, Mo M, Zhou SL, Wu J (2016). Long non-coding RNA metastasis associated in lung adenocarcinoma transcript 1 (MALAT1) interacts with estrogen receptor and predicted poor survival in breast cancer. Oncotarget.

[16] Chang L, Yuan Y, Li C, Guo T, Qi H, Xiao Y, Dong X, Liu Z, Liu Q (2016). Upregulation of SNHG6 regulates ZEB1 expression by competitively binding miR-101-3p and interacting with UPF1 in hepatocellular carcinoma. Cancer Lett.

[17] Su S, Gao J, Wang T, Wang J, Li H, Wang Z (2015). Long non-coding RNA BANCR regulates growth and metastasis and is associated with poor prognosis in retinoblastoma. Tumour Biol.

[18] Chen X, Kong J, Ma Z, Gao S, Feng X (2015). Up regulation of the long non-coding RNA NEAT1 promotes esophageal squamous cell carcinoma cell progression and correlates with poor prognosis. Am J Cancer Res.

[19] Chakravarty D, Sboner A, Nair SS, Giannopoulou E, Li RH, Hennig S, Mosquera JM, Pauwels J, Park K, Kossai M, MacDonald TY, Fontugne J, Erho N (2014). The oestrogen receptor alpha-regulated lncRNA NEAT1 is a critical modulator of prostate cancer. Nat Commun.

[20] Yu KD, Shen ZZ, Shao ZM (2009). The immunohistochemically “ER-negative, PR-negative, HER2-negative, CK5/6-negative, and HER1-negative” subgroup is not a surrogate for the normal-like subtype in breast cancer. Breast Cancer Res Treat.

[21] Endo Y, Dong Y, Kondo N, Yoshimoto N, Asano T, Hato Y, Nishimoto M, Kato H, Takahashi S, Nakanishi R, Toyama T (2016). HER2 mutation status in Japanese HER2-positive breast cancer patients. Breast Cancer.

[22] Jerry DJ, Dunphy KA, Hagen MJ (2010). Estrogens, regulation of p53 and breast cancer risk: a balancing act. Cell Mol Life Sci.

[23] Ding X, Zhu L, Ji T, Zhang X, Wang F, Gan S, Zhao M, Yang H (2014). Long intergenic non-coding RNAs (LincRNAs) identified by RNA-seq in breast cancer. PLoS One.

[24] Muppirala UK, Honavar VG, Dobbs D (2011). Predicting RNA-Protein Interactions Using Only Sequence Information. Bmc Bioinformatics.

[25] Chuang SC, Scélo G, Lee YC, Friis S, Pukkala E, Brewster DH, Hemminki K, Tracey E, Weiderpass E, Tamaro S, Pompe-Kirn V, Kliewer EV, Chia KS (2010). Risks of second primary cancer among patients with major histological types of lung cancers in both men and women. Br J Cancer.

[26] Quadrelli S, Lyons G, Colt H, Chimondeguy D, Silva C (2009). Lung cancer as a second primary malignancy: increasing prevalence and its influence on survival. Ann Surg Oncol.

[27] Yan Y, Zhang L, Jiang Y, Xu T, Mei Q, Wang H, Qin R, Zou Y, Hu G, Chen J, Lu Y (2015). LncRNA and mRNA interaction study based on transcriptome profiles reveals potential core genes in the pathogenesis of human glioblastoma multiforme. J Cancer Res Clin Oncol.

[28] Rosa FE, Silveira SM, Silveira CGT, Bergamo NA, Neto FAM, Domingues MAC, Soares FA, Caldeira JRF, Rogatto SR (2009). Quantitative real-time RT-PCR and chromogenic in situ hybridization: precise methods to detect HER-2 status in breast carcinoma. Bmc Cancer.

[29] Amaral PP, Clark MB, Gascoigne DK, Dinger ME, Mattick JS (2011). lncRNAdb: a reference database for long noncoding RNAs. Nucleic Acids Res.

[30] Chen G, Wang Z, Wang D, Qiu C, Liu M, Chen X, Zhang Q, Yan G, Cui Q (2013). LncRNADisease: a database for long-non-coding RNA-associated diseases. Nucleic Acids Res.

[31] Cerami E, Gao J, Dogrusoz U, Gross BE, Sumer SO, Aksoy BA, Jacobsen A, Byrne CJ, Heuer ML, Larsson E, Antipin Y, Reva B, Goldberg AP (2012). The cBio Cancer Genomics Portal: An Open Platform for Exploring Multidimensional Cancer Genomics Data (vol 2, pg 401, 2012). Cancer Discov.

[32] Cui D, Yu CH, Liu M, Xia QQ, Zhang YF, Jiang WL (2016). Long non-coding RNA PVT1 as a novel biomarker for diagnosis and prognosis of non-small cell lung cancer. Tumour Biol.

[33] Tuo YL, Li XM, Luo J (2015). Long noncoding RNA UCA1 modulates breast cancer cell growth and apoptosis through decreasing tumor suppressive miR-143. Eur Rev Med Pharmacol Sci.

[34] Chou J, Wang B, Zheng T, Li X, Zheng L, Hu J, Zhang Y, Xing Y, Xi T (2016). MALAT1 induced migration and invasion of human breast cancer cells by competitively binding miR-1 with cdc42. Biochem Biophys Res Commun.

[35] Zeng Z, Bo H, Gong Z, Lian Y, Li X, Li X, Zhang W, Deng H, Zhou M, Peng S, Li G, Xiong W (2016). AFAP1-AS1, a long noncoding RNA upregulated in lung cancer and promotes invasion and metastasis. Tumour Biol.

[36] Iden M, Fye S, Li KG, Chowdhury T, Ramchandran R, Rader JS (2016). The lncRNA PVT1 Contributes to the Cervical Cancer Phenotype and Associates with Poor Patient Prognosis. Plos One.

[37] Takahashi Y, Sawada G, Kurashige J, Uchi R, Matsumura T, Ueo H, Takano Y, Eguchi H, Sudo T, Sugimachi K, Yamamoto H, Doki Y, Mori M, Mimori K (2014). Amplification of PVT-1 is involved in poor prognosis via apoptosis inhibition in colorectal cancers. Br J Cancer.

[38] Wu Q, Guo L, Jiang F, Li L, Li Z, Chen F (2015). Analysis of the miRNA-mRNA-lncRNA networks in ER+ and ER- breast cancer cell lines. J Cell Mol Med.

[39] Li X, Zhang S, Liu W, Li H (2014). The effect of tamoxifen on expression of ER, PR, Cerb-B2, and ki-67 in C3H mice spontaneous breast cancer model and the relation with chemotherapeutic effect. Cell Biochem Biophys.

[40] Smits AJ, Kummer JA, Hinrichs JW, Herder GJ, Scheidel-Jacobse KC, Jiwa NM, Ruijter TE, Nooijen PT, Looijen-Salamon MG, Ligtenberg MJ, Thunnissen FB, Heideman DA, de Weger RA, Vink A (2012). EGFR and KRAS mutations in lung carcinomas in the Dutch population: increased EGFR mutation frequency in malignant pleural effusion of lung adenocarcinoma. Cell Oncol (Dordr).

[41] Ueda M, Iguchi T, Nambara S, Saito T, Komatsu H, Sakimura S, Hirata H, Uchi R, Takano Y, Shinden Y, Eguchi H, Masuda T, Sugimachi K (2015). Overexpression of Transcription Termination Factor 1 is Associated with a Poor Prognosis in Patients with Colorectal Cancer. Ann Surg Oncol.

[42] Thunnissen E, Boers E, Heideman DA, Grünberg K, Kuik DJ, Noorduin A, van Oosterhout M, Pronk D, Seldenrijk C, Sietsma H, Smit EF, van Suylen R, von der Thusen J (2012). Correlation of immunohistochemical staining p63 and TTF-1 with EGFR and K-ras mutational spectrum and diagnostic reproducibility in non small cell lung carcinoma. Virchows Arch.

[43] Edge SB, Compton CC (2010). The American Joint Committee on Cancer: the 7th Edition of the AJCC Cancer Staging Manual and the Future of TNM. Ann Surg Oncol.

[44] Schmittgen TD, Livak KJ (2008). Analyzing real-time PCR data by the comparative C(T) method. Nat Protoc.

