# Supplementary material for: Long non-coding RNAs may serve as biomarkers in breast cancer combined with primary lung cancer
Source: Oncotarget. 2017 Apr 21;8(35):58210–21. doi: 10.18632/oncotarget.17356 (PMC5601645; doi:10.18632/oncotarget.17356)
Supplement: Supplementary file 1 [file oncotarget-08-58210-s001.pdf]

Long non-coding RNAs may serve as biomarkers in breast cancer combined with primary lung cancer

Supplementary Materials

Supplementary Table 1: The results of lncRNA screening from qRT-PCR in breast cancer using lncRNA profiler based on lncRNAdb. See Supplementary_Table_1

Supplementary Table 2: The results of lncRNA screening from qRT-PCR in breast cancer using lncRNA profiler based on lncRNAdisease. See Supplementary_Table_2

Supplementary Table 3: The results of lncRNA screening from qRT-PCR in lung cancer using lncRNA profiler based on lncRNAdb. See Supplementary_Table_3

Supplementary Table 4: The results of lncRNA screening from qRT-PCR in lung cancer using lncRNA profiler based on lncRNAdisease. See Supplementary_Table_4

Supplementary Table 5: The primers information of selected lncRNAs for qRT-PCR

lncRNA	Primer	Sequence primers	Length/nt
AFAP1-AS1	Forward primer	5′ -AGGGCCTCTTTCCTCTTCAG-3′	20
	Reverse primer	5′- CCATGCCTGGCTAATTTTGT-3′	20
aHIF	Forward primer	5′-TTGTGGCTACCACGTACTGC -3′	20
	Reverse primer	5′-GGGAGTTTATCCCTTTTTCG-3′	20
BDNF-AS	Forward primer	5′-GTTCTTCGGGAATGTGGCTA-3′	20
	Reverse primer	5′-TTGGCTGTTTTGACTCGATG-3′	20
Kucg1	Forward primer	5′-GTGTGGCAACAACTCTGTGC-3′	20
	Reverse primer	5′-GCAGTTTGAATGTCCCTGGT-3′	20
Malat1	Forward primer	5′-CTCCCCACAAGCAACTTCTC-3′	20
	Reverse primer	5′-TTCAACCCACCAAAGACCTC-3′	20
BANCR	Forward primer	5′-ACCTGTGATCTCTGGCTGCT -3′	20
	Reverse primer	5′-TGTGCTCTTTGCAGAGGTG-3′	20
EXOC3-AS1	Forward primer	5′-CTCTCCTTTCCCTTCCCAAC-3′	20
	Reverse primer	5′-AACCTTCTCCGCTTTGGTTT-3′	20
PVT1	Forward primer	5′-TAGATCCTGCCCTGTTTGCT-3′	20
	Reverse primer	5′-CTTCAGGCCTCTTTGACAGC-3′	20
LINC00467	Forward primer	5′-TCGTCTTCAGGAAGCCAGC-3′	20
	Reverse primer	5′-TGGAAATCAAAAGGGTCAGC-3′	20
HCG18	Forward primer	5′-CCACAGGTACTGGTGGTGTG-3′	20
	Reverse primer	5′-CAAGCTCCAAGGATCTCTGC-3′	20
SNHG6	Forward primer	5′-AGGTGCAAGAAAGCCTTTGA-3′	20
	Reverse primer	5′-GCATGCCACACTTGAGGTAA-3′	20
LINC00657	Forward primer	5′-AAAGAGGTTGCCGACGTATG-3′	20
	Reverse primer	5′-CAGGTCTTCCAGCTCCATGT-3′	20
